# Case Report: *IGFBP5*-*ALK* fusion-positive case of high-grade endometrial stromal sarcoma with response to ALK-targeted therapy

**DOI:** 10.3389/fonc.2025.1720854

**Published:** 2026-01-21

**Authors:** Weiwei Zhang, Jinkun Wu, Shujie Song, Yu Cheng

**Affiliations:** 1 Department of Medical Oncology, The Affiliated Yantai Yuhuangding Hospital of Qingdao University, Yantai, Shandong, China; 2 Department of Pathology, The Affiliated Yantai Yuhuangding Hospital of Qingdao University, Yantai, China

**Keywords:** ALK-targeted therapy, high-grade endometrial stromal sarcoma, IGFBP5-ALK fusion, iruplinalkib, molecular profiling

## Abstract

**Background:**

High-grade endometrial stromal sarcoma (HG-ESS) is a very rare and aggressive uterine malignancy. Although recurrent genetic alterations such as *YWHAE*-*NUTM2* fusions and *BCOR* alterations are well recognized, *ALK* rearrangements have not been previously reported in HG-ESS, and the efficacy of ALK inhibitors in this context remains unknown.

**Case description:**

A 51-year-old woman presented with irregular vaginal bleeding and underwent a total abdominal hysterectomy with bilateral salpingo-oophorectomy (TLH/BSO) and omentectomy. Histopathological and immunohistochemical analyses following hysterectomy confirmed HG-ESS. Postoperative imaging revealed rapid disease progression with pulmonary and pelvic metastases. After failure of gemcitabine/docetaxel chemotherapy, next-generation sequencing identified an *IGFBP5*-*ALK* fusion (breakpoint: *IGFBP5* exon 1 – *ALK* exon 19), a *TERT* promoter mutation, and a homozygous *CDKN2A/CDKN2B*/*MTAP* deletion. The patient received iruplinalkib, a second-generation ALK inhibitor, and achieved a partial response within six weeks, with a >47.2% reduction in target lesions. The patient remains on therapy to date and treatment was well-tolerated.

**Conclusion:**

This case highlights the first documented response to an ALK inhibitor in *ALK*-rearranged HG-ESS. The findings underscore the importance of comprehensive molecular profiling in identifying targetable alterations in rare sarcomas and support the use of iruplinalkib as an effective therapeutic option in this setting.

## Introduction

Endometrial stromal sarcomas (ESS) are rare mesenchymal malignancies accounting for less than 1% of all uterine malignancies and approximately 10% of uterine sarcomas (1). The World Health Organization (WHO) classifies ESS into low-grade endometrial stromal sarcoma (LG-ESS), high-grade endometrial stromal sarcoma (HG-ESS) and undifferentiated uterine sarcoma based on histopathological and molecular features (2). HG-ESS is a distinct entity that typically exhibits more aggressive behavior, characterized by high-grade cytological features, frequent recurrence, and metastasis (3). Molecularly, HG-ESS is heterogeneous, with recurrent genetic alterations including *YWHAE*-*NUTM2* fusions, *BCOR* genetic alterations (rearrangements or internal tandem duplications), and many more being revealed as the next-generation sequencing (NGS) technique is being advanced (4, 5).

Conventional treatment for localized HG-ESS involves surgical resection, but management of advanced or metastatic disease remains challenging. Chemotherapy regimens, such as doxorubicin-based regimens or gemcitabine/docetaxel, are often used but with limited efficacy and significant toxicity. Given the poor outcomes with conventional chemotherapy, targeted therapies directed against specific molecular alterations represent a promising approach.

ALK inhibitors, such as crizotinib, alectinib, iruplinalkib and lorlatinib, have demonstrated remarkable efficacy in *ALK*-rearranged non-small cell lung cancer (NSCLC) (6–9). There is emerging evidence that ALK inhibitors may also be active in *ALK*-rearranged inflammatory myofibroblastic tumors (IMTs) and other sarcomas (10, 11). However, experience with ALK inhibitors in *ALK-*rearranged HG-ESS is limited. Iruplinalkib is a novel, potent second-generation ALK tyrosine kinase inhibitor approved by the Chinese National Medical Products Administration for ALK-positive NSCLC that has shown high overall response rates and improved outcomes compared with crizotinib in clinical trials (6). However, its use in *ALK*-rearranged sarcomas, particularly uterine sarcomas, has not been reported.

Herein, we present a case of metastatic HG-ESS harboring an *IGFBP5-ALK* fusion that demonstrated a dramatic and rapid response to iruplinalkib after progression on chemotherapy, highlighting the importance of molecular profiling and the therapeutic potential of ALK inhibitors in this rare malignancy.

## Methods

Written consent was obtained from the patient and results from genetic profiling and clinical data can be published.

### Histological analysis

Surgical resection specimens of uterine HG-ESS were fixed in 10% neutral buffered formalin, embedded in paraffin, and sectioned at a thickness of 5 μm. The sections were subsequently subjected to hematoxylin and eosin (H&E) staining and immunohistochemically evaluated using antibodies against ALK, CD10, H-caldesmon, Vimentin, ER, PR, P53, WT-1, CyclinD1, BRG1, INI-1, SMA, Desmin, CD117, S-100 and Ki67.

### Next-generation sequencing analysis

Genomic profiling was performed on DNA extracted from the patient’s formalin-fixed, paraffin-embedded (FFPE) tumor tissue sample. Targeted NGS was carried out using the BGI-T7 platform and a 506-gene panel encompassing cancer-related genes. Raw sequencing data in BCL format were converted to FASTQ files using bcl2fastq (v2.19). Quality control was performed with Trimmomatic to remove low-quality reads and reads containing N bases. Clean reads were then aligned to the hg19 human reference genome using BWA. Subsequent variant calling and annotation were performed using bioinformatics pipelines to detect point mutations, insertions/deletions, copy number alterations, and gene fusions, as well as tumor mutation burden (TMB) and microsatellite instability (MSI) status.

## Case description

**Figure 1:**
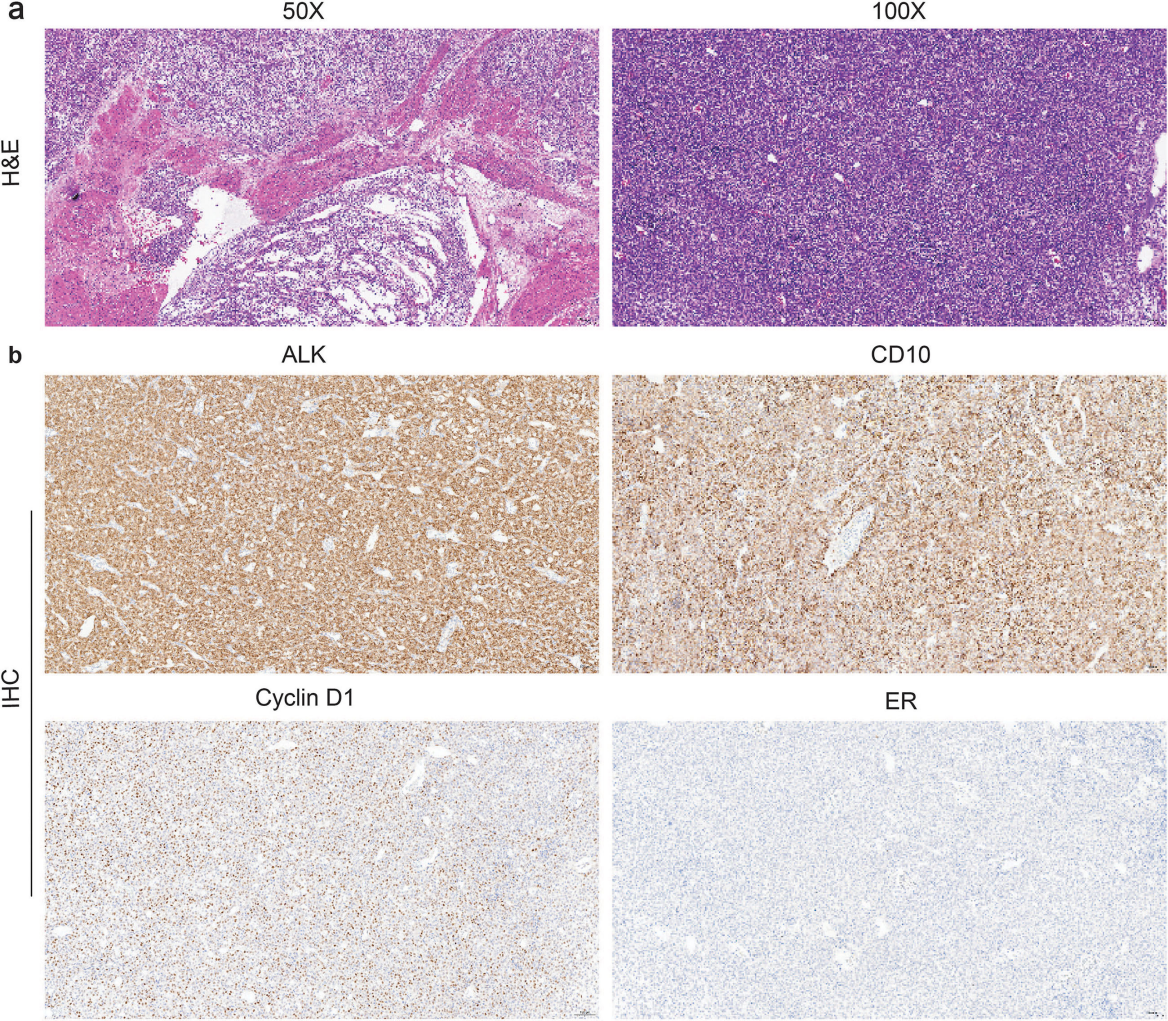
Morphologic features and immunohistochemical stains of the HG-ESS samples. **(a)** Representative pictures of H&E staining. **(b)** IHC showing expression of ALK, CD10, Cyclin D1, and ER. HG-ESS, high-grade endometrial stromal sarcoma; H&E, hematoxylin and eosin; IHC, immunohistochemistry.

A 51-year-old woman presented in September 2024 with irregular vaginal bleeding for one month. On 4 September 2024, she underwent a total abdominal hysterectomy with bilateral salpingo-oophorectomy (TLH/BSO) and omentectomy. The excised mass measured 15 × 11 × 9 cm and was located predominantly within the myometrium, with serosal penetration and lymphovascular invasion. No adnexal involvement was identified. Histopathological examination revealed a multinodular growth pattern with destructive invasion of the myometrium. The neoplastic cells were arranged in solid sheets, exhibited a high nuclear-to-cytoplasmic ratio, and possessed ovoid to plump spindle-shaped nuclei with prominent nucleoli; intravascular tumor emboli were also observed (**Figure 1a**). Immunohistochemically, the tumor cells demonstrate positivity for CD10, Cyclin D1, Vimentin and ALK (D5F3 clone), negativity for Desmin, CD117, ER, PR, and S-100, and a high proliferative index (Ki-67: 60%) (**Figure 1b**). These morphological and immunohistochemical features are consistent with a diagnosis of HG-ESS. According to the intraoperative findings and final postoperative pathology report from the hysterectomy specimen, the patient’s disease was classified as FIGO (2023) stage IVB.

**Figure 2:**
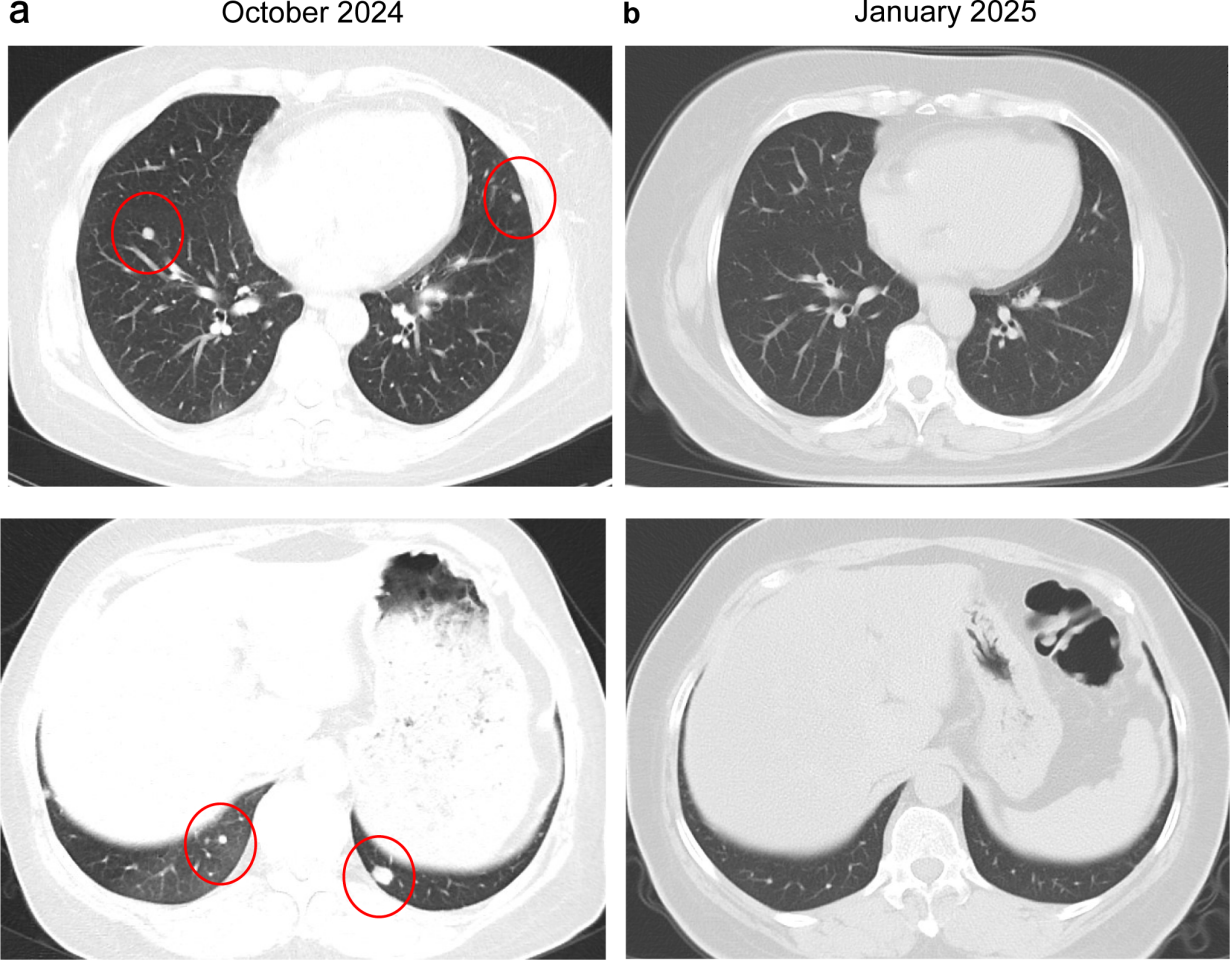
Chest CT scans before and after first-line chemotherapy. **(a)** Pre-chemotherapy CT image (October 25, 2024) demonstrates multiple bilateral pulmonary metastases. **(b)** Post-chemotherapy CT scan (January 13, 2025) shows significant remission of the pulmonary metastases. CT, computed tomography.

**Figure 3:**
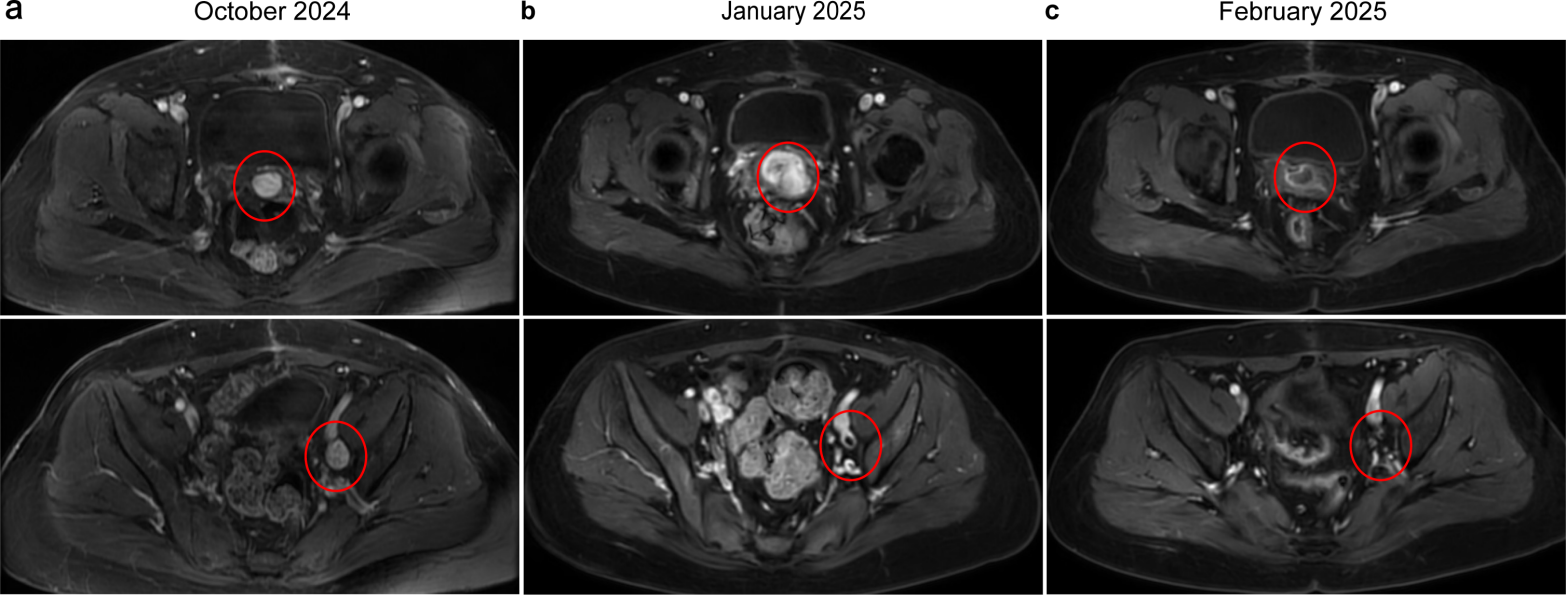
Baseline and follow-up contrast-enhanced MRI of the abdomen and pelvis. **(a)** Pre-chemotherapy images (October 25, 2024) reveal a new metastatic pelvic mass posterior to the bladder along with pelvic lymphadenopathy. **(b)** After chemotherapy (January 13, 2025), the pelvic lymph node metastases show shrinkage; however, the metastatic lesion behind the bladder exhibits 90% enlargement. **(c)** Following the initiation of Iruplinalkib treatment, both the pelvic metastatic lesion and lymph node metastases demonstrate a significant radiographic response (February 24, 2025).

Approximately one month (October 25, 2024) after surgery, surveillance imaging confirmed disease progression. Chest CT demonstrated multiple bilateral pulmonary metastases (**Figure 2a**). Contrast-enhanced abdominal and pelvic MRI revealed a new metastatic pelvic mass posterior to the bladder and progressive enlargement of pelvic lymphadenopathy, suggestive of metastatic nodal involvement (**Figure 3a**). The patient was subsequently treated with docetaxel and gemcitabine with progression of disease after two cycles. Follow-up chest CT (January 13, 2025) demonstrated a reduction in pulmonary metastases (**Figure 2b**). However, contrast-enhanced abdominal and pelvic MRI showed shrinkage of pelvic lymph node metastases but a 90% enlargement of the pelvic metastatic lesion posterior to the bladder, indicating localized disease progression (**Figures 3b**). Chemotherapy-induced anemia and fatigue made further chemotherapy intolerable. NGS was performed using a targeted 506-gene panel. The results identified an *IGFBP5-ALK* gene fusion, a *TERT* promoter mutation, and a homozygous deletion at chromosome 9p21.3 affecting the *CDKN2A*/*CDKN2B*/*MTAP* gene cluster (**Table 1**). No canonical molecular alterations typically associated with HG-ESS, such as *YWHAE*-*NUTM2* fusions or BCOR rearrangements/internal tandem duplications, were detected. Additionally, the tumor was found to be microsatellite stable and exhibited a TMB of 0 mutations per megabase.

**Table 1:** Genetic alterations detected by NGS.

Microsatellite stable
Tumor Mutational Burden = 0 mutations/megabase
*IGFBP5*: exon1-*ALK*: exon 19 fusion
*TERT* promoter (c.-124C>T) variant allele frequency = 28.95%
*CDKN2A* Homozygous deletion (CN 0.7)
*CDKN2B* Homozygous deletion (CN 0.66)
*MTAP* Homozygous deletion (CN 0.61)
*TNFRSF14* Copy Number Loss (CN 1.05)

In December 2024, the patient was started on an oral ALK inhibitor, iruplinalkib, at a dose of 180 mg once daily after a 7-day lead-in period at 60 mg once daily. After 6 weeks of treatment, follow-up abdominal and pelvic MRI (February 24, 2025) showed significant regression of all lesions (both the pelvic metastatic lesion posterior to the bladder and pelvic lymph node metastases) (**Figure 3c**). A partial response was observed based on RECIST 1.1 criteria, with the sum of the longest diameters of target lesions decreasing by more than 47.2%. The most recent follow-up examination on August 6, 2025, revealed further shrinkage of the metastatic lesion behind the bladder on contrast-enhanced abdominal and pelvic MRI. Local therapy for the pelvic recurrence was evaluated and administered. Following discussion by our multidisciplinary tumor board, the patient received a course of definitive intensity-modulated radiotherapy (IMRT) to the pelvis, targeting the recurrent pelvic lesion and involved pelvic lymph nodes. The treatment was delivered from September 22 to October 29, 2025. The planning target volumes and prescribed doses were as follows: the gross tumor volume of nodal disease (GTVn) with a 5-mm margin (PGTVn) received 61.6 Gy in 28 fractions, and the clinical target volume (CTV) encompassing the pelvic nodal drainage areas with a 5-mm margin (PTV) received 50.4 Gy in 28 fractions. The patient continues to receive iruplinalkib and remains under routine clinical surveillance. The treatment was generally well-tolerated, with only grade 1 hypertriglyceridemia reported.

## Discussion

In this case report, we present a case of a 51-year-old patient diagnosed with a HG-ESS and a very rare *IGFBP5*-*ALK* fusion. This report provides the first evidence of successful ALK-directed therapy in *ALK*-rearranged HG-ESS, achieving a significant and sustained response (>8 months and ongoing) following failure of conventional chemotherapy. This finding provides critical clinical evidence that *ALK*-rearranged HG-ESS represents a distinct molecular subtype for which targeted therapy with ALK inhibitors can be highly effective, mirroring the paradigm established in *ALK*-positive lung cancer.

ALK inhibitors, such as crizotinib, alectinib, iruplinalkib and lorlatinib, have demonstrated remarkable efficacy in *ALK*-rearranged NSCLC (6–9). There is a paucity of data on the incidence of *ALK* rearrangements and the role of ALK-directed tyrosine kinase inhibitors (TKIs) in the management of HG-ESS. Of note, the *IGFBP5*-*ALK* fusion identified in this HG-ESS (**Table 1**) differs from canonical NSCLC ALK rearrangements (e.g., *EML4*-*ALK*). NGS results also revealed the rearrangement breakpoint was between IGFBP5 exon 1 and ALK exon 19. The finding of this atypical IGFBP5-ALK breakpoint (the majority of ALK gene fusions occur at exon 20 of ALK) suggests that it may not be an isolated event, and highlights the great diversity of fusion events involving HG-ESS. The activity of *ALK* fusions may differ depending on the fusion partner and rearrangement breakpoints (12). Kyi et al. reported a patient with uterine inflammatory myofibroblastic tumor (IMT) positive for *IGFBP5*-*ALK* was shown to respond to crizotinib, however the rearrangement breakpoint was between *IGFBP5* exon 1 and *ALK* exon 20 (13). Another patient with uterine IMT achieved a complete response to crizotinib, although the rearrangement breakpoint was unknown (14). This patient’s response to iruplinalkib suggests that the I*GFBP5*-*ALK* (I1; A19) fusion is likely ALK inhibitor–sensitive. *ALK* fusions are exceedingly rare in HG-ESS, and this case identifies a novel initial driver event as demonstrated by the presence of I*GFBP5*-*ALK* fusion in this patient’s hysterectomy specimen and a paucity of other genetic alterations in the NGS results.

The genomic landscape was further defined by a homozygous deletion at the *CDKN2A/CDKN2B/MTAP* locus, which is recurrent alterations in HG-ESS and is strongly associated with aggressive clinical behavior and poor prognosis (15, 16). A retrospective molecular analysis of NSCLC patients with *ALK* rearrangements revealed that *CDKN2A/B* copy number loss was the second most frequent concomitant genetic alteration after TP53 mutation (17). In addition to *CDKN2A/B* copy number loss, co-occurring *TERT* gene mutations have been identified in the study (17). Interestingly, this patient-who was found to have *CDKN2A/B* copy number loss-exhibited a low TMB (0 mutations/Mb), which is inconsistent with the study showing that co-occurrence of *CDKN2A/B* copy number loss is associated with a higher TMB (17), indicating *CDKN2A/B* loss might be a late event in tumor evolution of HG-ESS. The aggressive clinical course, marked by rapid postoperative recurrence, aligns with a separate study linking co-occurring *CDKN2A/B* loss to poorer progression-free and overall survival, underscoring its role in driving aggressive tumor biology in *ALK*-rearranged HG-ESS (18).

This case report describes a rare instance of HG-ESS harboring an *IGFBP5* -*ALK* gene fusion—a genetic alteration with major therapeutic and prognostic implications. To our knowledge, this specific fusion has not been previously reported in HG-ESS and is more characteristically associated with uterine IMT (19, 20). The use of NGS for fusion detection and the patient’s marked response to *ALK*-targeted therapy represent significant strengths of this study. A limitation of our molecular analysis is that the sequencing data were processed using a customized bioinformatics pipeline designed to detect a predefined set of variants and fusions. Although this targeted approach ensures high sensitivity for known alterations, it may lack the breadth to identify novel or unexpected genetic events that could further inform the tumor’s biology or clinical behavior. As a single case report, our study has inherent limitations. The findings describe the experience of one patient and therefore cannot establish the general efficacy of ALK inhibitors in *ALK*-rearranged HG-ESS.

In conclusion, this case strengthens the rationale for molecular testing, including ALK immunohistochemistry and/or NGS, in patients with HG-ESS. The identification of an *ALK* rearrangement can transform the treatment strategy from ineffective chemotherapy to a highly effective, well-tolerated targeted therapy. Iruplinalkib, as demonstrated here, represents a promising treatment option for this molecularly defined subset of patients. Further clinical trials are warranted to formally establish the efficacy of ALK inhibitors in *ALK*-rearranged HG-ESS.

## Data Availability

The original contributions presented in the study are included in the article/supplementary material. Further inquiries can be directed to the corresponding author/s.
